# Prospective biomarker study in newly diagnosed glioblastoma: Cyto-C clinical trial

**DOI:** 10.1093/noajnl/vdab186

**Published:** 2021-12-24

**Authors:** Corinne E Griguer, Claudia R Oliva, Christopher S Coffey, Merit E Cudkowicz, Robin A Conwit, Anna L Gudjonsdottir, Dixie J Ecklund, Janel K Fedler, Tina M Neill-Hudson, Louis B Nabors, Melanie Benge, James R Hackney, Marianne Chase, Timothy P Leonard, Toral Patel, Howard Colman, Macarena de la Fuente, Rekha Chaudhary, Karen Marder, Teri Kreisl, Nimish Mohile, Milan G Chheda, Katharine McNeill, Priya Kumthekar, Aclan Dogan, Jan Drappatz, Vinay Puduvalli, Agnes Kowalska, Jerome Graber, Elizabeth Gerstner, Stephen Clark, Michael Salacz, James Markert

**Affiliations:** 1 Department of Radiation Oncology, University of Iowa, Iowa City, Iowa, USA; 2 Department of Biostatistics, University of Iowa, Iowa City, Iowa, USA; 3 Department of Neurology, Massachusetts General Hospital, Harvard Medical School, Boston, Massachusetts, USA; 4 NINDS, National Institutes of Health, Bethesda, Maryland, USA; 5 Department of Neurosurgery, University of Alabama at Birmingham, Birmingham, Alabama, USA; 6 Department of Neurology, University of Alabama at Birmingham, Birmingham, Alabama, USA; 7 Department of Pathology, Division of Neuropathology, University of Alabama at Birmingham, Birmingham, Alabama, USA; 8 Division of Neuro-Oncology, Columbia University Health Sciences, New York, New York, USA; 9 Feinberg School of Medicine, Northwestern University, Chicago, Illinois, USA; 10 Department of Neuro-Oncology, Ohio State University, Columbus, Ohio, USA; 11 Department of Neurosurgery, Oregon Health and Science University, Portland, Oregon, USA; 12 Department of Neurology, State University of New York, Stony Brook, New York, New York, USA; 13 Alvord Brain Tumor Center, Swedish Medical Center, Seattle, Washington, USA; 14 Department Internal Medicine, University of Cincinnati Medical Center, Cincinnati, Ohio, USA; 15 School of Medicine, University of Miami, Miami, Florida, USA; 16 Department of Medicine, University of Pittsburgh Medical Center, Pittsburgh, Pennsylvania, USA; 17 Department of Neurological Surgery, University of Texas Southwestern Medical Center, Dallas, Texas, USA; 18 Department of Neurosurgery, University of Utah, Salt Lake City, Utah, USA; 19 Departments of Medicine and Neurology, Washington University School of Medicine, St. Louis, Missouri, USA; 20 Montefiore Medical Center, Bronx, New York, USA; 21 Department of Neurology, Vanderbilt University, Nashville, Tennessee, USA; 22 Department Internal Medicine, University of Kansas Hospital, Kansas City, Kansas, USA

**Keywords:** biomarker, cytochrome C oxidase, glioblastoma, *MGMT*, prospective clinical trial

## Abstract

**Background:**

Glioblastoma (GBM) has a 5-year survival rate of 3%-5%. GBM treatment includes maximal resection followed by radiotherapy with concomitant and adjuvant temozolomide (TMZ). Cytochrome C oxidase (CcO) is a mitochondrial enzyme involved in the mechanism of resistance to TMZ. In a prior retrospective trial, CcO activity in GBMs inversely correlated with clinical outcome. The current Cyto-C study was designed to prospectively evaluate and validate the prognostic value of tumor CcO activity in patients with newly diagnosed primary GBM, and compared to the known prognostic value of *MGMT* promoter methylation status.

**Methods:**

This multi-institutional, blinded, prospective biomarker study enrolled 152 patients with newly diagnosed GBM who were to undergo surgical resection and would be candidates for standard of care. The primary end point was overall survival (OS) time, and the secondary end point was progression-free survival (PFS) time. Tumor CcO activity and *MGMT* promoter methylation status were assayed in a centralized laboratory.

**Results:**

OS and PFS did not differ by high or low tumor CcO activity, and the prognostic validity of *MGMT* promoter methylation was confirmed. Notably, a planned exploratory analysis suggested that the combination of low CcO activity and *MGMT* promoter methylation in tumors may be predictive of long-term survival.

**Conclusions:**

Tumor CcO activity alone was not confirmed as a prognostic marker in GBM patients. However, the combination of low CcO activity and methylated *MGMT* promoter may reveal a subgroup of GBM patients with improved long-term survival that warrants further evaluation. Our work also demonstrates the importance of performing large, multi-institutional, prospective studies to validate biomarkers. We also discuss lessons learned in assembling such studies.

**Key Points**

- The NeuroNext NN106 (Cyto-C) study was the first prospective blinded biomarker trial conducted in glioblastoma patients, and applied many components of the prospective-specimen collection, retrospective-blinded evaluation (PRoBE) design.
- The level of CcO had no prognostic value on overall survival or progression-free survival in glioblastoma.
- The combination of low CcO activity and *MGMT* promoter methylation status may be predictive of long-term survival and warrants further study.

**Importance of the Study**

To the best of our knowledge, we conducted, the first clinical trial to prospectively evaluate a prognostic biomarker in primary GBM. The clinical trial incorporates biomarker-relevant (non-interventional) elements of a “PRoBE” design, including blinding. A significant strength of our study was the diversity of the study sample (race, sex, age) and geographic reach (19 sites across the United States). Multi-institutional biomarker studies that require rapid tissue acquisition and processing, prior to centralized review, are feasible with a multidisciplinary team. A high percentage of patients consented to having their tissue studied, even prior to surgery and definitive diagnosis. While the level of CcO was not validated to have prognostic value on overall survival, exploratory analysis showed that low CcO activity and *MGMT* promoter methylation in GBMs may be a predictive marker for longer survival. Additional prospective cohorts are needed to confirm our findings and determine the utility of this potential combination of biomarkers.

Glioblastoma (GBM) is the most aggressive primary brain tumor. Even with the current standard of care (SOC) treatment, which includes radiation and concomitant and adjuvant temozolomide (TMZ),^1,2^ the median survival time for patients with primary GBM is only 14 months.^3^ This poor survival time may be due, in part, to the heterogeneity observed in GBM from different patients and even within primary and recurrent tumors.^4,5^ This heterogeneity suggests that unique treatment strategies may be necessary for subsets of GBMs. Among the many potential biomarkers assessed in GBM, methylation of the *O*^6^-methylguanine (*O*^6^-meG) DNA methyltransferase gene (*MGMT*) promoter is a reliable predictor of a favorable clinical response to TMZ.^1^ Beyond *MGMT* promoter methylation status and despite extensive efforts, a molecular signature that can be used as a prognostic or predictive marker in GBM remains lacking.^6,7^ Among the many potential biomarkers assessed in GBM, methylation of *MGMT* promoter is a reliable predictor of a favorable clinical response to TMZ.^1^ Recently, we also observed that cytochrome C oxidase (CcO, complex IV; EC 1.9.3.1) activity level associated with the acquisition of chemoresistance to TMZ in malignant gliomas.^8,9^

CcO is the terminal enzyme of the mitochondrial respiratory chain (electron transport chain [ETC]) that catalyzes the transfer of electrons from cytochrome C (Cyt *c*) to oxygen (O_2_), and thus regulates the electron flux capacity of the ETC. Of particular relevance to GBM therapy, high CcO activity supports more efficient mitochondrial coupling and thus decreased the production of reactive oxygen species (ROS),^8–11^ thereby diminishing the efficacy of chemotherapeutic drugs such as TMZ.^12,13^ In our previous retrospective study, high CcO activity was correlated with poor overall survival (OS) and progression-free survival (PFS).^14^ A receiver-operating characteristic (ROC) analysis in that study determined that a CcO/citrate synthase (CS) ratio of 4 was the optimal cutoff value. Given that such a biomarker would allow the identification of subsets of patients that exhibit similar characteristics, we set out to confirm the prognostic value of CcO activity status (high CcO/CS > 4 vs low CcO/CS ≤ 4) in a prospective, blinded, multicenter trial (the Cyto-C study), and compare its utility as a biomarker with that of *MGMT* promoter methylation status.

## Materials and Methods

### Trial Organization

The Cyto-C study was cooperatively developed and implemented by the Network for Excellence in Neuroscience Clinical Trials (NeuroNEXT), which is sponsored by the National Institute of Neurological Disorders and Stroke (NINDS), Grant number U01 NS093663. The trial was designed by a protocol working group and managed by a protocol steering committee, clinical coordinating center (CCC), and data coordinating center (DCC) and underwent scientific review and NINDS Council approval. Cyto-C study is the sixth trial to be conducted by the network and the first in neuro-oncology. Key aspects of this trial were the prequalification of the clinical sites, centralized ethics oversight, and master clinical trial agreements. Importantly, each site volunteered to participate, affirmatively stating their interest and ability to enroll and conduct the trial at their site. The 19 sites participating in this trial were academic medical centers, which agreed to participate in a centralized IRB review process prior to involvement in NeuroNEXT.

### Patients, Treatment, and Follow-up

The study consisted of an enrollment phase followed by a 24-month follow-up phase wherein the primary endpoint was OS and the secondary endpoint was PFS. Subjects with a potential diagnosis of a brain tumor were consented for tissue collection prior to initial surgical resection. Key eligibility criteria included subjects ≥21 years of age, a newly diagnosed GBM centrally confirmed at the University of Alabama at Birmingham (UAB), and availability of tumor tissue representative of GBM following tumor resection. Key exclusion criteria were secondary GBM or other gliomas, planned upfront treatment with any anti-angiogenic agent targeting the vascular endothelial growth factor pathway, or any immunotherapy regimen. Complete details regarding inclusion and exclusion criteria are provided in Supplementary file 1. Subjects consented to allow excess brain tumor tissue to be sent for central review, analysis and to allow study staff to collect information from their clinical records. Once consent was obtained, the diagnosis of primary GBM was confirmed by on-site neuropathologists and centrally confirmed at UAB. All subjects received maximal safe resection followed by radiation with concomitant TMZ, then maintenance TMZ chemotherapy as prescribed by their treating physician. The SOC follow-up schedule was per each institution’s typical SOC protocol. While on therapy, subjects were evaluated in clinic or via telephone interviews at 3-month intervals from the time of surgery. At the end of 24 months or at the time of death, the site completed an exit form that specified treatment details and time of tumor progression. Each subject’s clinical records were reviewed by study staff who extracted pertinent data for the study.

### Tissue Acquisition and Processing

Brain tumor tissue was collected in the operating room of each clinical site during surgery. Approximately 70 mg of fresh tissue was collected per subject and immediately transferred to sterile ice-cold PBS, then snap-frozen in liquid nitrogen and labeled with the patient ID number allocated by the Cyto-C DCC. Clinical sites were provided with a standard operating procedure (SOP) and 7-min video describing in detail the procedures for tumor tissue sample collection, processing samples, and shipping of the samples. All the individuals involved in tissue collection were required to watch the video and read the SOP, then sign a Training Log which was sent to the DCC for review and record. A study kit containing the proper items needed for sample storage and shipment was sent to the sites in order to assure standardization of the tissue collection process between sites. All evaluable specimens were snap-frozen within 30 minutes of resection and spent 10 minutes or less at room temperature. Snap-frozen tissue specimens were immediately stored at −80°C until they were shipped overnight to the central laboratory for the Cyto-C study at UAB. After receipt by the central laboratory, tissue specimens were stored at −80°C until they were processed for CcO and CS activity and *MGMT* promoter methylation status determination. The detailed SOP is provided in Supplementary file 2.

### Mitochondrial Isolation

Isolation of mitochondria from GBMs was performed as previously described.^14^ Briefly, each tumor specimen was weighed, minced, and suspended in ice-cold isolation buffer (250 mM sucrose, 10 mM Tris-HCl, 0.5 mM EDTA; pH 7.4), then manually homogenized. The homogenate was centrifuged for 5 minutes at 1000 × *g*, and the pellets (nuclear enriched fractions) were frozen prior to DNA isolation. The supernatants were centrifuged for 10 minutes at 12 500×*g* to obtain enriched mitochondrial pellets, which were stored at −80°C prior to CcO activity determination. Mitochondria were subsequently solubilized in 10 mM potassium phosphate buffer supplemented with 0.2% *n*-dodecyl β-d-maltoside (LM) and protease/phosphatase inhibitors, extracted on ice for 1 hour, and centrifuged at 10 000×*g* for 10 minutes. Supernatant protein concentration was determined by the Bradford assay.

### Enzymatic Activity

The reliability of the CcO and CS activity assays was assessed by estimating precision and accuracy according to ISO standard 5725-1. Series were compared using Wilcoxon-rank and Spearman-rank tests. These assays were performed by the same operator using 2 different machines.

Spectrophotometric determination of CS (EC 4.1.3.7), a Krebs cycle enzyme also found in the mitochondria, and CcO activity levels were performed as previously described.^8,14,15^ Briefly, CS activity was measured at 415 nm in potassium phosphate buffer, pH 7.2, with the addition of 2.5 mM dithionitrobenzoic acid, 2.5 mM acetyl-CoA, and 10 mM oxaloacetate. The increase in absorbance was used to calculate CS enzyme activity. CcO activity was measured in potassium phosphate buffer, pH 7.2, with the addition of 10 µM reduced Cyt *c*. The oxidation of Cyt *c* was measured as the decrease in absorbance at 550 nm and was used to calculate CcO enzyme activity. CcO activity was expressed as micromoles of Cyt *c* oxidized per second per mg protein. The activity of CS remains stable in isolated mitochondria. Therefore, CS activity was used to normalize CcO activity.^16–18^

### DNA Isolation

Purified DNA was obtained from the nuclear enriched fraction of each tumor using the QIAamp DNA Kit (Qiagen # 51306) according to the manufacturer’s instructions. The genomic DNA yields, concentration, and A260/A280 ratio were determined using a NanoDrop spectrometer based on the A260 and A280 readings. The genomic DNA integrity was analyzed on 1% agarose gel.

### Methylation-Specific PCR

*MGMT* promoter methylation patterns were determined by chemical modification of unmethylated cytosines to uracil and subsequent PCR using primers specific for either methylated or unmethylated DNA, according to Esteller et al.^19^ DNA was treated with sodium bisulfite as previously described.^20^ Briefly, 4 µg of genomic DNA was incubated with 0.3 M NaOH at 50°C for 20 minutes to denature the DNA. The mixture was then incubated for 20 hours at 50°C in 500 µL of a freshly prepared solution containing 3 M sodium bisulfite and 10 mM hydroquinone. DNA was subsequently purified with a Wizard DNA Clean-Up System (Promega, Madison, WI, USA), following the instructions of the manufacturer, then resuspended in 100 µL of deionized H_2_O and stored at −80°C until use. Primer sequences for the unmethylated *MGMT* promoter were: 5′-TTTGTGTTTTGATGTTTGTAGGTTTTTGT-3′ (forward primer) and 5′-AACTCCACACTCTTCCA AAAACAAAACA-3′ (reverse primer). Primer sequences for the methylated *MGMT* promoter were: 5′-TTTCGACGTTCGTAGGTTTTCGC-3′ (forward primer) and 5′-GCACTCTTCCGAAAAC GAAAC G-3′ (reverse primer).^19^ Optical signals of the methylated and unmethylated PCR products were quantified with ImageJ as previously described.^21^

### Statistical Analysis

Study personnel assessing tumor progression were blinded to the participant’s CcO activity status. As determined in the prior retrospective study, tumors with CcO/CS ratio scores below or equal to 4 were categorized as having low CcO activity, while tumors with ratio scores above 4 were categorized as having high CcO activity. The full analysis population consisted of all centrally confirmed eligible participants and was used to address the primary objective and all analyses looking at OS. A SOC population was defined as the subset of participants in the full analysis population for whom death occurred within 12 weeks of surgery or SOC could be confirmed (any amount of radiation therapy or concomitant TMZ starting within 12 weeks from the date of surgery or if bevacizumab/avastin was taken it must have occurred 6 weeks after radiation and/or concomitant TMZ for patients under 65 and within 3 weeks for patients 65 and over). The SOC population was used for all analyses examining PFS as the endpoint. The significance threshold was set at a 2-sided *P*-value of <.05 for all analyses.

The primary outcome was OS, defined as the time from the day of surgery until death from any cause. At the time of final analysis, the OS of any subject still alive after 24 months from diagnosis was treated as a right-censored observation. The secondary outcome, PFS, was defined as the time from the day of surgery for the primary tumor until tumor recurrence, as detected by clinical and radiographic evidence of progression according to RANO criteria,^22^ or death from any cause. For patients who were alive at the time of analysis and did not show progression at the time of their last routine visit, PFS was treated as a right-censored observation.

The OS and PFS survival functions were estimated using the Kaplan-Meier estimator, and the median survival times and corresponding 95% confidence interval (CI) were reported. Log-rank tests were used to compare survival between the high vs low CcO tumor activity groups. Cox proportional hazards regression models were used to estimate the hazard of the OS and PFS events in the high CcO tumor activity group relative to the low CcO group. The proportional hazards assumption was assessed by examining a plot of Schoenfeld residuals against survival time. The percentage of deaths, which is the percentage of participants that had an event within a given group of interest during their time on study, was summarized for OS analyses. A Wilcoxon weighted log-rank test and Fisher’s exact test comparing the proportion of participants experiencing an event in each group were conducted separately for OS and PFS as sensitivity analyses. The effect of *MGMT* promoter methylation status on OS and PFS was assessed using similar methods.

The sample size for the primary and secondary objectives was computed based on the log-rank test. We assumed a 2-year event rate of 95% and 30% of participants displaying high tumor CcO activity levels. For the primary aim, 145 evaluable subjects provided 95% power to detect a hazard ratio of 1.85 using a 2-sided log-rank test. Results of the power analysis indicated the study would be adequately powered to detect clinically meaningful effects for a reasonable range of anticipated CcO frequencies.

In addition, a multivariate Cox proportional hazards regression model was used to study the simultaneous effects of CcO activity level and *MGMT* promoter methylation status as part of a preplanned exploratory analysis. The model included main effect terms for both CcO activity level and *MGMT* promoter methylation status, as well as an interaction term to determine if the effect of *MGMT* promoter methylation status was moderated by CcO activity level. It was recognized that this approach will provide reasonable power to detect a large interaction, but a nonsignificant test of interaction cannot be interpreted to suggest the absence of interaction. Thus, we further explored post hoc subgroup analyses to examine the role of methylation status within each CcO activity level.

## Results

### Study Cohort

The study was registered on ClinicalTrials.gov (NCT02997423). Central IRB approval for the first site was obtained on October 31, 2016, with the final site approved on July 11, 2017. The first subject was enrolled in December 2016, and the last subject was enrolled in June 2020. In all, 259 subjects were consented at an average rate of 0.83 per site per 30 days. Because subjects were consented before a final diagnosis of primary GBM, only 152 matched the eligibility criteria. The main reasons for ineligibility were (1) subjects not meeting inclusion criteria (81 of 259); (2) inadequate tissue (40 of 259); (3) GBM not confirmed (36 of 259); and (4) subjects meeting exclusion criteria (34 of 259). Altogether, 104 of the 259 subjects enrolled failed screening. In addition, 1 patient declined to participate and 2 patients had unevaluated tissue samples that precluded a central confirmation of eligibility. Among the final study cohort, approximately half of the subjects were male and most subjects identified as White (Table 1). Mean age was 61.0 ± 11.1 years.

**Table 1.** Study Demographics and Clinical Characteristics

	High Tumor CcO Activity (N = 68)	Low Tumor CcO Activity (N = 84)	Total (N = 152)	*P*-value
Gender				
Male	38 (55.9%)	41 (48.8%)	79 (52%)	.42
Female	30 (44.1%)	43 (51.2%)	73 (48%)	
Ethnicity				
Hispanic or Latino	4 (7.4%)	9 (13.0%)	13 (10.6%)	.38
Non-Hispanic or Latino	50 (92.6%)	60 (87.0%)	110 (89.4%)	
Unknown/not reported	14	15	29	
Race				
White	50 (87.7%)	61 (89.7%)	111 (88.8%)	.78
Non-White	7 (12.3%)	7 (10.3%)	14 (11.2%)	
Unknown/not reported	11	16	27	
Receipt of SOC				
Yes	60 (88.2%)	78 (92.9%)	138 (90.7%)	.40
No	8 (11.8%)	6 (7.1%)	14 (9.2%)	
*MGMT* status-methylated^a^	11 (19%)	22 (32%)		.15
Height (cm)^b^	169 (10.5)	170 (10.5)		.59
Weight (kg)	82 (16.2)	86 (21.8)		.21
Age at surgery	61 (11.6)	61 (10.8)		.85
Karnofsky score	82 (11.4)	82 (9.5)		.85

Abbreviations: CcO, cytochrome C oxidase; SOC, standard of care.

^a^ *MGMT* status was “undetermined” for 26 participants.

^b^Height missing for 1 participant.

### OS and PFS by Tumor CcO Activity

We first determined the levels of CcO and CS activity in mitochondria isolated from the primary GBM tissue specimens. CS activity was used to normalize CcO activity in each sample. The mean CcO/CS ratio for the entire population was 4.40 ± 2.89 (minimum, 0.17; maximum, 15.6; n = 152). The distribution of CcO/CS activity did not differ significantly based on demographic and clinical characteristics, including sex; ethnicity; race; height, weight, and age at surgery; and Karnofsky score (Table 1). We were able to measure CcO and CS activity in all 152 eligible specimens.

**Figure 1.**
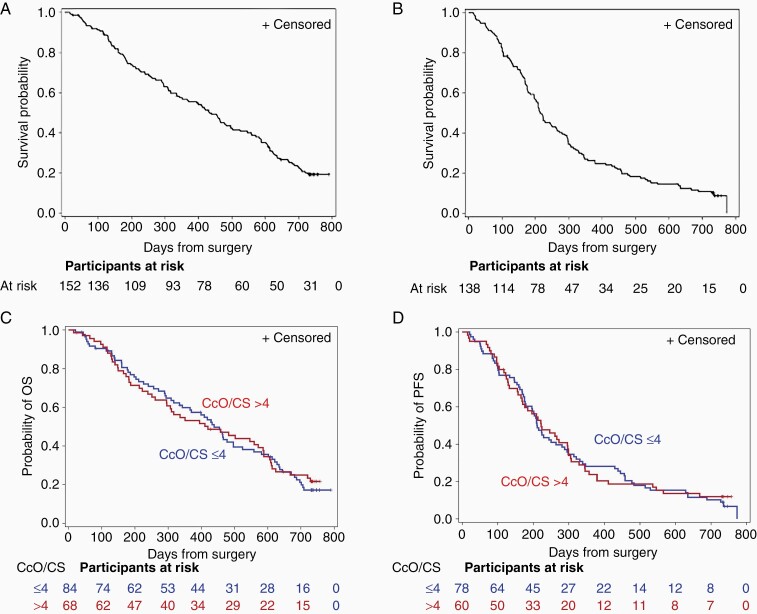
Kaplan-Meier survival curves in patients with newly diagnosed GBM. (A, B) Median OS (A) and median PFS (B) in the full cohort. (C, D) Median OS (C) and PFS (D) in patients stratified by tumor CcO/CS activity. Low tumor CcO activity (blue line) was defined as CcO/CS ≤ 4 and high tumor CcO activity (red line) was defined as CcO/CS > 4. Abbreviations: CcO, cytochrome C oxidase; CS, citrate synthase; GBM, glioblastoma; OS, overall survival; PFS, progression-free survival.

For the entire cohort, median OS was 432 (95% CI, 336 to 502) days, and median PFS was 215 (95% CI, 196 to 264) days (Figure 1A and B). When patients were stratified by tumor CcO activity level (high vs low), neither OS nor PFS differed significantly between the 2 groups. High tumor CcO activity was detected in 68 patients (45%) and low tumor CcO activity was detected in 84 patients (55%). The median OS was 438 (95% CI, 334 to 496) days among patients with low tumor CcO activity and 420 (95% CI, 295 to 586) days among patients with high tumor CcO activity (*P* = .84 by log-rank test) (Figure 1C). The hazard ratio for death was 0.96 (95% CI, 0.67 to 1.39). The median PFS was 211 (95% CI, 177 to 281) days among patients with low tumor CcO activity and 222 (95% CI, 168 to 297) days among patients with high tumor CcO activity (*P* = .85 by log-rank test) (Figure 1D).

### OS and PFS by *MGMT* Promoter Methylation Status

**Figure 2.**
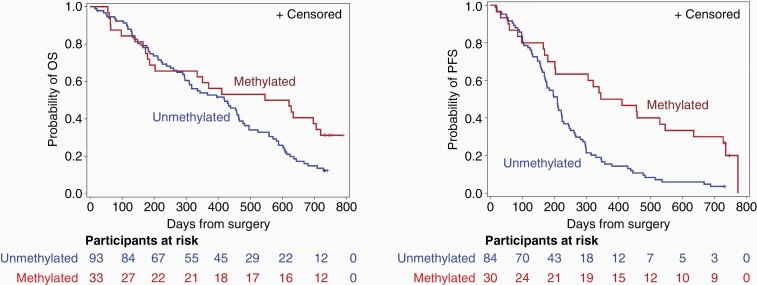
Kaplan-Meier survival curves in patients with newly diagnosed GBM stratified by tumor *MGMT* promoter methylation status. (A) Median OS by *MGMT* promoter methylation status. (B) Median PFS by *MGMT* promoter methylation status. Abbreviations: GBM, glioblastoma; OS, overall survival; PFS, progression-free survival.

The secondary objective of the study was to compare the ability of CcO activity level to prognose OS and PFS in GBM with the ability of *MGMT* promoter methylation status. Tumor *MGMT* promoter methylation status was undetermined for 26 participants. When patients were stratified by *MGMT* promoter methylation status (methylated vs unmethylated), OS and PFS each differed significantly between the 2 groups. For the OS analysis, *MGMT* promoter methylation status was determined in the tumors of 126 subjects, of which 33 had methylated promoters and 93 had unmethylated promoters. In the group with methylated promoters, 22 (67%) died, compared to 77 (83%) in the group with unmethylated promoters. The median OS was 584 (95% CI, 186 to 720) days among patients in the methylated *MGMT* promoter group, compared with 420 (95% CI, 296 to 464) days among those in the unmethylated *MGMT* promoter group (*P* = .043 by log-rank test) (Figure 2A). The hazard ratio for death was 0.61 (95% CI, 0.38 to 0.99). Because the sensitivity analyses were not consistent with Fisher’s exact test or Wilcoxon weighted log-rank test (*P* = .08 and *P* = .22, respectively), a modified Cox model was used to allow estimated hazard ratios to differ before and after 1 year. The hazard ratio for death before 1 year was 0.88 (95% CI, 0.47 to 1.65), and after 1 year was 0.40 (95% CI, 0.19 to 0.84). For the PFS analysis, *MGMT* promoter methylation status was established in 114 subjects, of which 30 had tumors with methylated promoters and 84 had tumors with unmethylated promoters. In the methylated *MGMT* promoter group, 24 subjects (80%) progressed; in the unmethylated *MGMT* promoter group, 81 subjects (96%) progressed. The median PFS was 378 (95% CI, 201 to 635) days among patients in the methylated *MGMT* promoter group, compared with 209 (95% CI, 170 to 224) days among those in the unmethylated *MGMT* promoter group (*P* < .001 by log-rank test) (Figure 2B).

### OS and PFS by Association of CcO Activity and *MGMT* Promoter Methylation Status

To explore the association between CcO activity level and *MGMT* promoter methylation status, tumors were stratified according to tumor CcO activity level (high or low) and *MGMT* promoter methylation status (methylated or unmethylated) in order to assess whether the effect of methylation status was modified by CcO activity group. Death occurred in 82% (9/11) in the methylated *MGMT* promoter/high CcO activity group, in 78% (36/46) in the unmethylated *MGMT* promoter/high CcO activity group, in 59% (13/22) in the methylated *MGMT* promoter/low CcO activity group, and in 87% (41/47) in the unmethylated *MGMT* promoter/low CcO activity group. A test of interaction between *MGMT* promoter methylation status and CcO activity did not meet the pre-specified threshold for significance (*P* = .067).

**Figure 3.**
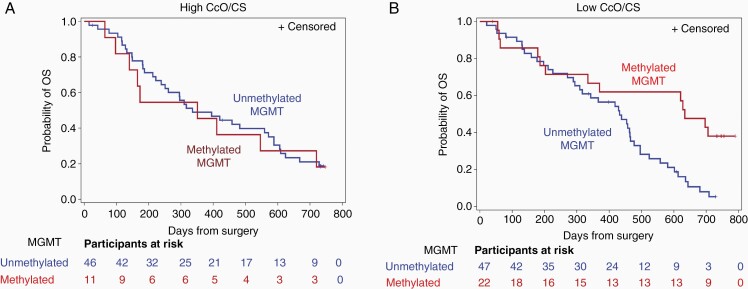
Median survival, log-rank, and Wilcoxon weighted log-rank test for OS by tumor CcO activity and *MGMT* promoter methylation subgroups. (A) Kaplan-Meier survival function estimate of OS in the high tumor CcO activity group by *MGMT* promoter status. (B) Kaplan-Meier survival function estimate of OS in the low tumor CcO activity group by *MGMT* promoter status. Abbreviations: CcO, cytochrome C oxidase; OS, overall survival.

We further explored post hoc subgroup analyses within each methylation status group. The hazard ratio of death for those with methylated vs unmethylated promoter status within the high CcO activity group was 1.07 (95% CI, 0.51 to 2.22; *P* = .86). Within the high CcO activity group, the median OS was 351 (95% CI, 97 to 720) days among the methylated *MGMT* promoters and 336 (95% CI, 239 to 571) days among the unmethylated *MGMT* promoters (Figure 3A). The hazard ratio of death for those with methylated vs unmethylated promoter status within the low CcO activity group was 0.43 (95% CI, 0.23 to 0.82; *P* = .01). The median OS was 634 (95% CI, 203 to undefined) days among the methylated *MGMT* promoter/low CcO activity group compared with 432 (95% CI, 294 to 466) days among the unmethylated *MGMT* promoter/low CcO activity group (*P* = .004 by log-rank test) (Figure 3B).

## Discussion

Efficient tumor-specific biomarkers are in high demand, as they are important in expediting diagnosis at initial stages, facilitating personalized treatment regimens, and providing helpful prognostic information for patients and their families. Despite the challenges faced in transforming the results of tumor-specific biomarker research into clinical practice, a large number of genomics- and proteomics-derived tumor markers have been proposed as diagnostic, prognostic, and/or predictive biomarkers of CNS tumors.^23^ However, numerous challenges have hindered the clinical transformation of preclinical findings regarding these potential biomarkers. To alleviate such challenges in our study of CcO activity status as a prognostic biomarker in GBM, we took advantage of the infrastructure and coordinated experience available through the NeuroNEXT Network. The Cyto-C study was designed to assess the clinical relevance of CcO activity as a prognostic biomarker in patients with newly diagnosed GBM. The Cyto-C trial was designed to ensure that specimens were collected according to a rigorous protocol, ensuring the documentation of factors that might influence biomarker values or disease characteristics, and to incorporate specimens from multiple centers. Cyto-C specimen sets thus have substantial value for use in the discovery phase of other potential GBM markers.^24^ The Cyto-C study has many components of a PRoBE design.^25^ Indeed, the Cyto-C study is the first in which biological specimens were collected prospectively from a single cohort of patients with newly diagnosed GBM, which represented the population envisioned for clinical application of the potential biomarker (ie, tumor CcO activity and *MGMT* promoter methylation status). This design excludes the common sources of bias found in case-control designs, where specimens are collected after the disease status is known. Underlining the importance of such studies, in this prospective biomarker trial, we did not confirm the prognostic value of CcO activity that was suggested in our previous discovery/retrospective study.^14^ The ratio used for this study was chosen based on the optimal cutoff based on a ROC analysis in that study. To further examine whether this cutoff affected the lack of validation, a set of exploratory analyses were conducted to evaluate the findings under alternative cutoff values. An analysis evaluating cutoff values ranging from 2.0 to 8.0 at 0.5 intervals revealed that the choice of cutoff values did not alter our final conclusions.

The lack of confirmation in the Cyto-C study may be related to the lack of standardization for tumor tissue collection and storage used in our previous study or the smaller sample size. In the Cyto-C study, all clinical sites adhered to the standard operating processes for tumor tissue collection and storage, allowing uniformity across all the sites. Other possible reasons for differences in outcomes in the Cyto-C study (vs our previous study) include the multicenter (vs single center) setting blinded (vs unblinded) outcomes analysis, pre-stipulated treatment and follow-up (vs no prospective stipulation of treatment or follow-up), and direct data collection (vs retrospective chart review). Another relevant difference may be the statistical design: the Cyto-C study was powered to validate CcO as a biomarker and both the statistical plan and analysis were developed before doing the assays for CcO activity and *MGMT* promoter methylation. In contrast, our retrospective study was not prospectively powered and the study design was confounded by specimen selection from a tumor tissue bank, which limited our selection and information regarding processing and storage protocols. It is also worth mentioning, while we were unable to validate CcO activity as a biomarker in the Cyto-C study, the prognostic value of *MGMT* promoter methylation status was again demonstrated, validating the overall design, marker assessment protocol, and data analysis and reporting in the Cyto-C study. This rigor was due to the coordinated efforts of the CCC, DCC, and the 19 clinical sites enrolling patients in this study.

In addition to suggesting that CcO activity is not a reliable prognostic biomarker in GBM, this study adds value to the field for several other reasons. First, we report a very high preoperative consent rate (99.1%). This means that patients on the cusp of a neurosurgical procedure, under stress of an unknown diagnosis, are not unapproachable. On the contrary, the high consent rate suggests they are very willing to provide excess tissue for research purposes. Second, our results demonstrate that coordination of multi-institutional biomarker studies in neuro-oncology, with centralized IRB procedures and requiring rapid tissue processing is feasible. This study highlights the importance of having infrastructures such as NeuroNEXT in place to easily and seamlessly perform prospective biomarker studies in brain tumors, with centralized analyses that require institution-based standardized processing.

A variety of molecular markers may have prognostic value in patients with GBM. These markers include high expression of *MGMT*, overexpression of *EGFR*, presence of *EGFR* vIII mutation, expression of the *YKL-40* gene, expression of tenascin-C, *PTEN* gene mutation or loss of function, loss of chromosome 10, and *p*53 gene mutation or loss of function.^1,17,26–28^ Although *MGMT* promoter methylation is well established as predicting tumor response to TMZ and patient outcomes, none of these markers had been definitively confirmed as a prospective biomarker in GBM treated with SOC. This trial prospectively confirmed the prognostic validity of *MGMT* promoter methylation status in GBM patients receiving current SOC treatment.^2,29^ Despite the survival benefit associated with *MGMT* promoter methylation, the OS curves remained similar for the first 12 months of follow-up, suggesting that patients with tumors bearing the methylated *MGMT* promoter have a better prognosis 12 months after the initial surgery. TMZ resistance has been associated with *MGMT* promoter methylation in several studies.^30–32^ However, some studies have indicated that this correlation does not hold true in all cases. Thus, Hegi et al^1^ concluded that there is no clear relationship between *MGMT* promoter methylation and a favorable response to TMZ treatment. These contradictory findings indicate the need to combine *MGMT* promoter methylation status with other biomarkers.^33^ Over the past decades, it has been clear that while *MGMT* methylation status has gained center stage, its specificity and selectivity performances are poor. Using CcO status upstream of *MGMT* methylation status could increase the performance of *MGMT* as a “true predictive marker” and significantly decrease the number of false-positive and false-negative cases and thus improve medical decisions and treatment strategy. We are beginning to understand a novel mechanism by which CcO drives resistance to TMZ/radiation. DNA damage and repair can be affected by cell metabolism in that the regulation of ROS through different metabolic pathways can increase oxidative damage to DNA.^7,34^ Epigenetic silencing of the *MGMT* gene by methylation of the promoter region has been shown to correlate with loss of expression of the *MGMT* protein resulting in decreased DNA repair.^2^ High levels of ROS in tumors harboring low CcO activity impose an extra burden for DNA repair.^7^ Low CcO activity (high ROS) and *MGMT* promoter methylation (lower DNA repair) may possibly explain why patients in this cohort had better outcomes following TMZ/radiation treatment. The results of this trial show that the combination of low CcO activity and *MGMT* promoter methylation may identify patients with GBM who are likely to be long-term survivors of SOC. Although tumor CcO activity alone was not confirmed as a prognostic marker in patients GBM, the interaction between CcO and methylated *MGMT* promoter warrants further evaluation.

## Supplementary Material

vdab186_suppl_Supplementary_Data_S1

vdab186_suppl_Supplementary_Data_S2
