# Supplementary material for: Prospective biomarker study in newly diagnosed glioblastoma: Cyto-C clinical trial
Source: Neurooncol Adv. 2021 Dec 24;4(1):vdab186. doi: 10.1093/noajnl/vdab186 (PMC8788017; doi:10.1093/noajnl/vdab186)
Supplement: vdab186_suppl_Supplementary_Data_S1 [file vdab186_suppl_supplementary_data_s1.docx]

NN106- KIT ITEM DESCRIPTION

1. Styrofoam Shipping Box
2. Cardboard Shipping Box
3. FedEX Shipping Label
4. FedEX Pouch
5. Dry Ice Shipping Sticker (UN1845; Class 9)
6. Exempt Human Specimen Sticker
7. Sharpie Pen
8. Biohazard Ziplock bag
9. Sterile Forceps
10. Specimen Cup pre-filled with sterile PBS and with sample size reference template
11. Cryo Vial

ITEMS NOT PROVIDED

1. Ice Bucket
2. Gloves
3. Liquid Nitrogen
4. Liquid Nitrogen Container
5. Dry Ice
6. Shipping tape

SCRIPT

BRIEF:

The goal of this video is to train NN sites how to process tissue samples from the OR, storage procedures and shipment procedures to parent site.

The video will provide information about:

1-Description of items included in the kit

2-Procedure of collection of sample from the Operating Room

3-Procedure of storage

4-Procedure for shipping on dry ice to parent site

1. Description of NN106 clinical trial kit
2. Styrofoam Box (1)
3. Cardboard Shipping Box (1)
4. FedEX Shipping Label Prefilled (1)
5. FedEX pouch (1)
6. Dry Ice Shipping Sticker (UN1845; Class 9) (1)
7. Exempt Human Specimen Sticker (1)
8. Sharpie Pen (1)
9. Biohazard Ziplock bag (1)
10. Pair of Sterile Forceps (1)
11. Specimen Cup pre-filled with sterile PBS and with sample size reference template
12. Cryo Vial with empty label to be filled with patient ID number (1)

ATTENTION: DO NOT DISCARD the shipping container including the Styrofoam box. You will need it for sending samples to parent site.

1. Procedure of collecting sample from the Operating Room at day of surgery

Before going to OR prepare:

1. An ice bucket filled with ice where you will place the specimen cup pre-filled with 20 ml PBS
2. A container filled with Liquid Nitrogen
3. The lid of the ice bucket
4. What to bring in the OR
5. Ice bucket filled with ice where you will place the specimen cup prefilled with 20 ml PBS
6. Place the lid on top of the ice bucket
7. Bring the sterile forceps
8. Pair of gloves (not provided)
9. In the OR
10. When the tumor has been resected and the sample allocated for NN106 trial is ready, take the sterile forceps and gently take the specimen and visually compared the size of the tissues with the sample size reference template located on top of the specimen cup lid. If the tissue sample is at least the size of the template, proceed to the next step.
11. Using the forceps provided in the kit gently drop the tissues into the cold PBS solution. Tightly and carefully close the lid. Return to the processing lab as soon as possible and (within 30 minutes of tumor resection).
12. In the processing lab, you should have in front of you: The container filled with liquid nitrogen, the cryo-vial previously labeled with the patient ID number with the sharpie provided in the kit, the specimen cup containing the tissues for the trial that you brought from the OR.

ATTENTION: The specimen cup containing the tissue should be kept on ice at all time

1. Remove the lid from the specimen cup and using the same forceps provided in the kit , gently take the tissue and gently drop inside the cryovial.
2. Close the lid of the cryo-vial tighly and drop it into the container pre-filled with liquid nitrogen.

Specimen is frozen when heavy bubbling stopped (1 minute)

1. Keep the cryo-vial containing frozen tissue inside the liquid nitrogen container till you reach the -80 C freezer

At this point the tissue is ready to be stored at -80 C.

ATTENTION: The cryo-vial MUST be labeled with the patient ID# before dropping into the liquid nitrogen.

1. Storage at -80 C.

To store tissue sample, remove the cryo-vial containing the frozen tissues from liquid nitrogen with forceps provided in the kit and quickly place at -80 C.

ATTENTION: The cryo-vial containing NN106 tissue sample MUST be kept at -80 C at all time and cannot be shipped till you received confirmation of Primary GBM diagnosis

At this point the specimen cup and the forceps can be discarded in biohazard waste

1. Shipping to parent site procedure

This step is only when diagnosed of Primary GBM has been confirmed.

You should have in front of you:

1. Styrofoam Shipping Box (1)
2. Cardboard Shipping Box (1)
3. FedEX Shipping Label Prefilled (1)
4. FedEX Pouch
5. Dry Ice Shipping Sticker (UN1845; Class 9) (1)
6. Exempt Human Specimen Sticker (1)
7. Sharpie Pen (1)
8. Biohazard Ziplock bag (1)

- Place the Styrofoam box inside the cardboard shipping box.
- Write in the name and address of shipper (your institution) and consignee ( parent site ) on the DRY ICE Label using sharpie pen provided in the kit
- Place Dry Ice shipping sticker (UN1845;Class 9) on the long side of the cardboard shipping box
- Place the “Exempt Human Specimen” sticker on the cardboard box next to the Dry Ice sticker
- Complete the sender info (Your Institution) on the FedEX shipping Label. The rest of the label will be pre-filled by Parent site
- Fill the Styrofoam shipping box half way with dry ice
- Quickly remove the cryo-vial from -80 C freezer and place it in the Biohazard Ziplock bag
- Fold the bag in half and in half again, and place it on top of dry ice
- Fill the rest of the Styrofoam shipping box with dry ice, leaving only enough space for the top to fit in place
- Place the top on the Styrofoam shipping box
- Seal the Cardboard shipping box using shipping tape ONLY (not provided). Tape the box along the seam with 3 strips of tape. Tape the ends with one strip of shipping tape.
- Tear off the Sender Copy (top copy) of the FedEX shipping label and retain it for your records
- Place FedEX shipping pouch on the top of the cardboard shipping box
- Place the remainder of the FedEX shipping label inside the FedEX pouch and seal the pouch
- YOUR BOX IS READY TO BE SENT

ATTENTION: Preparation of the Styrofoam box containing dry ice SHOULD BE DONE no more than 2 HRS before FedEX pick up

ATTENTION: Shipping only on MONDAY, TUESDAY and WESNESDAY. If you can NOT ship during these days, the tissues sample should stay at -80 C and NOT SHIPPED till the next week
