# Supplementary material for: Prospective biomarker study in newly diagnosed glioblastoma: Cyto-C clinical trial
Source: Neurooncol Adv. 2021 Dec 24;4(1):vdab186. doi: 10.1093/noajnl/vdab186 (PMC8788017; doi:10.1093/noajnl/vdab186)
Supplement: vdab186_suppl_Supplementary_Data_S2 [file vdab186_suppl_supplementary_data_s2.docx]

## Inclusion Criteria

***Subject Characteristics:***

1. Willingness and ability to provide written informed consent and to comply with the study protocol as judged by Physician interview (NOTE: This could be patient’s Neurosurgeon, Neuro-Oncologist or Study Investigator) or, if the patient lacks the capacity to provide informed consent, a legally authorized representative (LAR) has provided written informed consent.
2. Age ≥ 21 years.
3. KPS score ≥ 60.
4. Subjects’ planned upfront treatment to be SOC with Radiotherapy and Temozolomide (i.e. TEMODAR) for histologically confirmed GBM at initial diagnosis
5. No history of other malignancies except adequately treated non-melanoma skin cancer, curatively treated *in situ* cancer of the cervix, or other curatively treated solid tumors with no evidence of disease for at least 5 years
6. No serious active infection (e.g., wound infection requiring parenteral antibiotics) or other serious underlying medical conditions that in the opinion of the investigator would compromise standard of care treatment
7. No other condition (e.g., psychological or geographical) that would preclude study compliance
8. An MRI that is consistent with a primary malignant glioma

***Disease Characteristics:***

# Histologically confirmed newly diagnosed Primary GBM before treatment using WHO classification criteria (A local pathology report constitutes adequate documentation of histology for initial study enrollment, however central pathology review will be required to confirm the diagnosis of GBM for final data analysis).

# Viability of tumor tissue representative of GBM > 70 mg, snap-frozen within 30 minutes of resection, 10 minutes or less at room temperature

# All subjects must have received maximal safe resection followed by standard radiation with concomitant Temozolomide taken during the course of radiation therapy.

## 4.2 Exclusion Criteria

Exclusion criteria are proposed that will exclude subjects with pre-existing co-morbidities that could contribute to pre-mature death (e.g., significant cardiovascular history), with non-included pretreated tumors occupying either intracranial and extra-axial space, significantly impaired neurological performance status (e.g., KPS>60), with glial tumors that are genomically distinct from primary GBM tumors (e.g., gliomas arising from a previously diagnosed lower grade than GBM) or those unable to complete the fundamental requirements of the study.

**Pre-surgery/Prior to consent:**

1. Inability to fulfill the requirements of the protocol
2. Secondary GBM or other gliomas.
3. History of sensitivity to Temodar.
4. Planned upfront treatment with any anti-angiogenic agent targeting the VEGF pathway including but not limited to bevacizumab, cediranib, vandetanib, sunitinib, pazopanib, aflibercept or sorafenib or any immunotherapy regimen.

**Post-surgery/Post-consent:**

1. Any severe post-operative infection or other complications that may significantly delay the initiation of brain tumor therapy, or other conditions that, in the opinion of the investigator, would compromise the subject’s ability to participate in the study.

**Note:** Use of GLIADEL wafers in combination with surgical resection is allowed if patient is to follow standard of care treatment i.e. TMZ and radiation. Also, the tumor tissue resection is to occur prior to the insertion of the GLIADEL wafer. **Use of GLIADEL wafers during surgery combined with only radiation therapy is exclusionary.**
